# Toxicity and protein composition of venoms of *Hottentotta saulcyi*, *Hottentotta schach* and *Androctonus crassicauda*, three scorpion species collected in Iran

**DOI:** 10.1002/vms3.593

**Published:** 2021-08-06

**Authors:** Ani Boghozian, Habibollah Nazem, Mohammad Fazilati, Seyed Hossein Hejazi, Mohammadreza Sheikh Sajjadieh

**Affiliations:** ^1^ Department of Biochemistry Payame Noor University Tehran Iran; ^2^ Department of Parasitology and Mycology School of Medicine Isfahan University of Medical Sciences Isfahan Iran; ^3^ Ph.D. Clinical Immunology, Nobel Medical Laboratory Isfahan Iran

**Keywords:** albumins, amino acids, chromatography, high pressure liquid, lethal dose 50, mice, scorpion venoms

## Abstract

**Background:**

Scorpion stings comprise a serious problem throughout the globe, especially in regions where they are more frequent. Despite a recent upsurge of interest in scorpion venoms by various research groups, there remain many challenges.

**Objective:**

Therefore, in this study, we aimed to study the toxicity and protein composition of venoms of *Hottentotta saulcyi*, *Hottentotta schach* and *Androctonus crassicauda*, three scorpion species collected in Iran.

**Materials and Methods:**

Scorpion species were collected from Esfahan farm scorpion company and maintained in the laboratory in containers that mimic their natural habitat. Venom was extracted from *A. crassicauda*, *H. schach* and *H. saulcyi* by electrical stimulation of 8 and 10 V. The toxicity of each venom was established by using four groups of male Swiss albino mice aged 2 months (weighting 18–20 g) for testing each dose of venom. One group was used as a control. Venom was injected into mice by subcutaneous route. Then, animals were monitored for 24 h and LD_50_ was estimated by the graphic method of Miller and Tainter. Thus, high‐performance liquid chromatography–tandem mass spectrometry (HPLC–MS/MS) method was used to determine amino acids in the venom, and protein concentrations were determined by the Biuret method.

**Results:**

LD_50_ of scorpion venoms by subcutaneous route was found to be 1.70 mg/kg b.w (*A. crassicauda*), 1.47 mg/kg b.w (*H. saulcyi*) and 0.85 mg/kg b.w (*H. schach*). *A. crassicauda*, *H. saulcyi* and *H. schach* contain 26, 30, and 31 amino acids, respectively. *A. crassicauda* contains low concentrations of alpha‐aminoadipic acid, beta‐aminoisobutyric acid, beta‐alanine and citrulline*. H. saulcyi* contains a concentration of hydroxylysine, whereas *H. schach* has no such concentration. *A. crassicauda* also had the highest levels of tyrosine and threonine. Only *A. crassicauda* venom contains a low proportion of proteins (14.80%) compared with those of *H. schach* (16.26%) and *H. saulcyi* (16.20%). Albumin content in the venoms was 11.7% (*H. saulcyi*), 5.4% (*H. schach*) and 4.4% (*A. crassicauda*).

**Conclusion:**

Scorpions venoms have a variable toxicity and an interesting composition in amino acids and proteins. Work on the development of anti‐venom is fundamental.

## Graphical abstract

This study could be useful for understanding the venom extraction and toxicity, the protein and amino acid diversities of three species of scorpions (*Hottentotta saulcyi, Hottentotta schach* and *Androctonus crassicauda*). The results from this study show that *H. saulcyi* and *H. schach* contain more amino acids and protein and have higher toxicity than *A. crassicauda*.

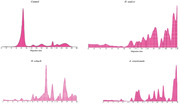

## 1 INTRODUCTION

Scorpions are arthropod species belonging to an ancient family of arachnids dating back almost 400 million years (Jeyaprakash & Hoy, 2009; Kazemi & Sabatier, 2019). They are represented by about 2200 species and are distributed geographically around the world (Dehghani et al., 2016; Ward et al., 2018). A total of 64 species of scorpions are distributed in Iran belonging to three families: Buthidae, Hemiscorpiidae and Scorpionidae (Motevalli Haghi & Dehghani, 2017; Navidpour et al., 2020). These species were found mostly in tropical regions of Iran (Motevalli Haghi & Dehghani, 2017).

Scorpions are venomous arachnids with major medical health importance in Iran (Navidpour et al., 2020). They have acquired the ability to defend against predators and capture prey by producing toxin‐laden venom secreted by specialised venomous glands found at the end of the scorpion telson (Sollod et al., 2005). Envenomation of scorpion is a public health problem worldwide and in particular in Iran (Yadav et al., 2020).

Throughout their long evolutionary existence on this planet, coupled with the selective pressure exerted on these organisms, the scorpions have succeeded in developing series of poisonous peptides that present various biological activities and pharmacological functions (de la Vega et al., 2010). Scorpions have always received attention in Iran because of their frequency and venom. High doses of venom can cause death and hypersensitive reactions (Saganuwan, 2018). In the past 50 years, many aspects of scorpion biology have been studied in Iran, including venom (Motevalli Haghi & Dehghani, 2017). Several scattered studies on the scorpion have been conducted, but the maintenance on the captivity of some species, venom extraction, toxicity and amino acid constituent is still not clear.

According to the literature, in Iran, *Androctonus* and *Hottentotta* genera contain dangerous and medically important species (Hauke & Herzig, 2017; Ward et al., 2018). *Hottentotta saulcyi* (*H. saulcyi*)*, Hottentotta schach* (*H. schach*) and *Androctonus crassicauda* (*A. crassicauda*) are three species of scorpions belonging to the family of Buthidae (Dehghani et al., 2009). They are among the species most involved in scorpion stings in Iran (Dehghani & Fathi, 2012). There are insufficient data on the toxicity of their venom and its amino acid and protein composition.

Studying the different methods of captivity, venom extraction, its toxicity and its constitution is of paramount importance for researchers. This work aims to study the toxicity and protein composition of venoms of *H. saulcyi*, *H. schach* and *A. crassicauda*, three scorpion species collected in Iran.

## 2 MATERIALS AND METHODS

### 2.1 Collection of the scorpions

Three species of medically important scorpions were collected from Esfahan farm scorpion company and maintained in the laboratory in containers that mimic their natural habitat. They were captured carefully using long forceps by holding the tip of their tail without harming the animal. Scorpions were then transferred into separate containers with the soil of about two inches in depth upon which small stones were placed.

The venom was extracted from *A. crassicauda, H. schach* and *H. saulcyi* by electrical stimulation (8–10 V).

### 2.2 Identification of scorpions

A stereomicroscope and a morphological identification key were used to identify the scorpion. Based on morphological characteristics such as body colour, pedipalp shape and size, sternum shape, telson colour and several pectin teeth, collected scorpions were identified at the species level.

### 2.3 Keeping of scorpions

In the laboratory, scorpions were placed in a large glass aquarium (diameter: 54 cm, height 22 cm) on top layer of soil about 3 in. in depth, above which proper size stones were kept, imitating their natural habitat.

As a source of drinking water, a petri dish lid containing tap water was kept in the centre of the glass aquarium. The scorpions were fed with crickets and worms of flour. At 3‐day intervals, the water content of the petri dish was checked and, if necessary, filled.

Scorpions were only handled on the day of venom extraction and during soil replacement, which was done once every 2 months. The venom was not removed the day immediately following the feed and no food was added up to 3 days after the venom was removed.

The tail was held firmly wing forceps and electrically stimulated by pointing two electrodes connected to a step‐down transformer at junctions between the tail segments, one next to the telson. The other at the junction between IV and V metasomal segment. *A. crassicauda*, *H. schach* and *H. saulcyi* were stimulated twice, at 8 and 10 V, respectively, for 2–3 s with short intervals between two stimulations.

The venom was allowed to secrete over a piece of parafilm placed at the base of its tail and then transferred to a vial. The venom obtained in a single episode from many scorpions of a species were pooled, mixed with excess double distilled water and centrifuged at 15,000 rpm for 20 min to remove the mucus; the supernatant was lyophilised and stored at 20°C until used for protein estimation and toxicity studies; venom was resuspended in phosphate‐buffered saline (PBS) PH7.

### 2.4 Lethality of scorpion venom

The toxicity of venom from *A. crassicauda*, *H. schach* and *H. saulcyi* was studied by estimating the lethal dose 50 (LD_50_) value under in vivo conditions. Four groups of 2‐month‐old Swiss male albino mice (weighing 18–20 g), each group containing four mice, were used to test each dose of venom for toxicity, and one group of five was used as a control. The mice were kept at room temperature where they had libitum access to rodent food and surface water throughout the experiment.

Four different doses covering the range of mortality from 0 to 100 % were tested. The test group mice were subcutaneously injected with four concentrations. *A. crassicauda* (1, 1.8, 2.6, 2.8 mg/kg b.w), H. schach (0.45, 0.90, 1.8, 2.2 mg/kg b.w) and H. saulcyi (1.2, 1.5, 1.8, 2.1 mg/kg b.w) scorpion venoms were dissolved in 0.2 ml of PBS. The control group was injected with an equivalent volume of PBS only. The animals were monitored for 24 h and the LD_50_ was calculated by the graphical method of Miller and Tainter (Randhawa, 2009).

### 2.5 Determination of amino acids

The high‐performance liquid chromatography–tandem mass spectrometry (HPLC–MS/MS) method is used to determine amino acids in the venom of three medically important scorpion species.

The HPLC–MS/MS system consists of an Agilent 1200 high‐performance liquid chromatography coupled with an Agilent 6430 series triple quadrupole mass spectrometer with an electrospray ionisation source. The chromatographic separation was achieved on a CORTECS C18 column (4.6 mm x 150 mm, 2.7 μm) and the column temperature was kept at 30°C. Mobile phases which consisted of 0.1% formic acid in water (A) and acetonitrile (B) were used in the following gradient elution method: 0–10 min, 10–85% B; 10–13 min, 85–95% B; 13–19 min, 95–95% B. The flow rate was set at 0.3 ml/min, and the injection volume was 10 μl. All the data were analysed by Mass Hunter workstation software (Agilent Technologies, Palo Alto, CA, USA).

The mass spectrometer was carried out in both positive and negative ionisation multiple reaction monitoring mode. The source parameters were as follows: the capillary voltage set at 300 V for positive ionisation mode and −300 V for negative ionisation mode, the drying gas temperature was 320°C, the flow was 11 L/min and nebulising gas pressure was 30 psi.

### 2.6 Liquid chromatography with tandem mass spectrometry

All reagents were of analytical grade; the solvents were of chromatographic purity and the water was purified by deionisation (Milli‐Q system; Millipore, Bedford, MA, USA). Most of the standards were supplied by Fluka–Sigma–Aldrich (St. Louis, MO, USA). A few specific standards were supplied by Extrasynthèse (Genay, France; flavonoid glucosides), Radian International (Austin, Texas, USA; opiates) and pharmaceutical laboratories participating in the Brazilian government DST/AIDS program. Ampholyte solutions in the pH range 6–8 and 3–10 were supplied by Amersham Pharmacia Biotech (Uppsala, Sweden) and a 4% methylcellulose stock solution was supplied by Applied Biosystems (Foster City, CA, USA).

### 2.7 Concentration of proteins

Protein concentrations were determined by the Biuret method. In this technique, peptide bonds in proteins produce a violet colour in the presence of alkaline copper, and the intensity of the colour is estimated with a spectrometer at 540 nm wavelength.

All experiments were conducted in capillary electrophoresis systems, either model P/ACE 5510 from Beckman Instruments (Fullerton, CA, USA) or model HP^3D^CE from Agilent Technologies. The P/ACE unit was equipped with a filter‐carrousel UV detector, while the HP^3D^CE unit was equipped with a diode array detector. Both systems had temperature control devices, maintained at 25–30°C and data acquisition and treatment software supplied by the manufacturer. Uncoated fused‐silica capillaries (Polymicro Technologies, Phoenix, AZ, USA) were used in all experiments except for the haemoglobin analyses, where a dimethylpolysiloxane coated fused‐silica capillary (DB‐1, J&W Scientific, Folsom, CA, U.S.A.) with dimensions of 27 cm total length, 20 cm effective length, 50 cm i.d. and 0.20 cm coating thickness was used. At the beginning of the day, regular capillaries were typically washed with a 1 mol L^–1^ NaOH solution (high pressure, 5 min), followed by deionised water (5 min) and electrolyte (30 min). In between runs, the capillaries were usually reconditioned by a flush with the electrolyte solution (high pressure, 2 min). At the beginning of the day, the coated capillary was conditioned by a flush of methanol followed by deionised water (1.38 × 10^2^ kPa for 10 min, each flush). In between runs, the coated capillary was just replenished with fresh ampholyte working solution (2 min flush). At the end of the day, the coated capillary was washed with deionised water, methanol and dried with a flush of nitrogen (1.38 × 10^2^ kPa, 5 min each flush).

### 2.8 Statistical analysis

No statistical analysis was performed.

## 3 RESULTS

### 3.1 Collection of the scorpion

*A. crassicauda, H. schach* and *H. saulcyi* were collected from Esfahan Farm Scorpion Company and maintained in the laboratory in containers that mimic their natural habitat. They were captured carefully using long forceps by holding the tip of their tail without harming the animal. Scorpions were then transferred into separate containers with the soil of about two inches in depth upon which small stones were placed. The venom was extracted from *A. crassicauda, H. schach* and *H. saulcyi* by electrical stimulation (8 and 10 V).

### 3.2 Identification of scorpions

Based on morphological characteristics such as body colour, pedipalp shape and size, sternum shape, telson colour and several pectin teeth, identified scorpions were presented in Table 1.

**TABLE 1:** Identification of scorpions

Scorpion species	Body colour	Pedipalp shape	Pedipalp size	Sternum shape	Telson colour	Pectin teeth number
*Androctonus crassicauda*	Blackish‐brown to black	Slender pedipalp with bulbous chela	15‐25 mm	Triangular	Black	28
*Hottentotta saulcyi*	Yellow to yellowish green	Pedipalp all segments uniformly coloured	75–120 mm	Triangular	Black	30
*Hottentotta schach*	Brown to black	Segmented	90–130 mm	Triangular	Black	32

### 3.3 Lethality of scorpion venom

Table 2 shows the recorded mortality rates caused by *H. saulcyi* venom in mice. Four doses were used for the experiment (1.2, 1.5, 1.8, 2.1 mg/kg b.w). At the end of the analyses, the LD_50_ obtained was 1.47 mg/kg b.w.

**TABLE 2:** Lethality of *H. saulcyi* (1.2, 1.5, 1.8, 2.1 mg/kg b.w)

Group	Dose (mg/kg b.w)	Volume of injection (ml)	Mice (death/total)	Percentage of death (%)	LD50 (mg/kg b.w)	Probit value
1	1.2	0.2	1/4	25	1.22	5.67448975
2	1.5	0.2	2/4	50	1.47	5
3	1.8	0.2	3/4	75	1.77	4.32551025
4	2.1	0.2	4/4	100	2.14	–

Table 3 shows the recorded mortality rates caused by *H. schach* venom on mice. Four doses were used for the experiment (0.45, 0.90, 1.8, 2.2 mg/kg b.w). At the end of the analyses, the LD_50_ obtained was 0.85 mg/kg b.w.

**TABLE 3:** Lethality of *H. schach* (0.45, 0.90, 1.8, 2.2 mg/kg b.w)

Group	Dose (mg/kg b.w)	Volume of injection (ml)	Mice (death/total)	Percentage of death (%)	LD50 (mg/kg b.w)	Probit value
1	0.45	0.2	1/4	25	0.48	5.67448975
2	0.90	0.2	2/4	50	0.85	5
3	1.8	0.2	3/4	75	1.50	4.32551025
4	2.2	0.2	4/4	100	2.65	–

Table 4 shows the recorded mortality rates caused by the venom of *A. crassicauda* on mice. Four doses were used for the experiment (1.2, 1.8, 2.6, 2.8 mg/kg b.w). At the end of the analyses, the LD_50_ obtained was 1.70 mg/kg b.w.

**TABLE 4:** Lethality of *Androctonus crassicauda* (1.2, 1.8, 2.6, 2.8 mg/kg b.w)

Group	Dose (mg/kg b.w)	Volume of injection (ml)	Mice (death/total)	Percentage of death (%)	LD50 (mg/kg b.w)	Probit value
1	1.2	0.2	1/4	25	1.25	4.32551025
2	1.8	0.2	2/4	50	1.70	5
3	2.6	0.2	3/4	75	2.33	5.67448975
4	2.8	0.2	4/4	100	3.18	–

The LD_50_ recorded for the tested venoms were 1.47 mg/kg for *H. saulcyi*, 0.85 mg/kg for *H. schach* and 1.70 mg/kg for *A. crassicauda*. The venom of *A. crassicauda* thus presented the lowest toxicity and the highest toxicity was recorded for *H. schach*.

### 3.4 Determination of amino acids and low concentration amino acid

None of the venoms studied contain argininosuccinic acid and sulfocysteine. The venom of *A. crassicauda* has few amino acids compared with the venom of *H. saulcyi* and *H. schach*. *A. crassicauda* venom does not contain allo‐isoleucine, cystathionine, homocitrulline, homocystine, hydroxylysine and hydroxyproline. Similarly, unlike *H. schach, H. saulcyi* does not contain traces of cystathionine and homocystin at all. *H. saulcyi* contains a concentration of hydroxylysine, whereas *H. schach* has no such concentration.

*A. crassicauda, H. saulcyi* and *H. schach* contain 26, 30 and 31 amino acids, respectively, of which 7, 11 and 12 are in low concentrations (Table 5).

**TABLE 5:** Low concentration amino acids

Amino acid	*Androctonus crassicauda* (μM)	*Hottentotta saulcyi* (μM)	*Hottentotta schach* (μM)
Allo‐isoleucine	0	0.2	0.2
Alpha‐aminoadipic acid	0.6	0.6	0.7
Argininosuccinic acid	0	0	0
Beta‐aminoisobutyric acid	0.6	0.4	2
Beta‐alanine	0.6	2.4	8.5
Citrulline	1.8	4.6	92.4
Cystathionine	0	0	0.1
Cystine	0.3	1	0.1
Gamma‐aminobutyric acid	0.6	1.4	2.6
Glycylproline	0.6	0.6	2.9
Homocitrulline	0	0.2	0.2
Homocystine	0	0	0.1
Hydroxylysine	0	0.2	0
Hydroxyproline	0	0.2	0.9
Sulfocysteine	0	0	0

### 3.5 High concentration amino acid

Except for serine, arginine, aspartic acid, glutamine and threonine, *H. schach* had the highest concentrations of amino acids in high concentrations. Despite these amino acids, *H. schach* had the highest concentrations for all amino acids compared with *H. schach* and *A. crassicauda. A. crassicauda* also had the highest levels of tyrosine and threonine (Table 6).

**TABLE 6:** High concentration of amino acids

Amino acid	*A. crassicauda* (μM)	*H. saulcyi* (μM)	*H. schach* (μM)
Valine	28.8	56.8	315.6
Alanin	25.8	85.4	191.9
Arginine	114	329.2	7.4
Aspartic acid	35.1	42.2	9.2
Glutamic acid	12	42.4	59.8
Glutamin	12.6	65.8	28.3
Glycin	63.9	287.6	422.7
Histidine	14.4	18.4	77.4
Isoleucine	19.8	82.2	1109
Leucine	217.2	1049.8	1638.6
Lysine	255.6	181	3445
Methionine	24	80.2	631.3
Ornithine	9	6.6	11.9
Phenylalanine	79.8	187.6	638.3
Proline	25.4	143.6	548.2
Serine	76.2	313.6	88
Threonine	116.1	70.4	13.5
Tryptophan	41.4	337.2	488.8
Tyrosine	108.3	80.4	1992.5

### 3.6 Concentration of proteins

**FIGURE 1:**
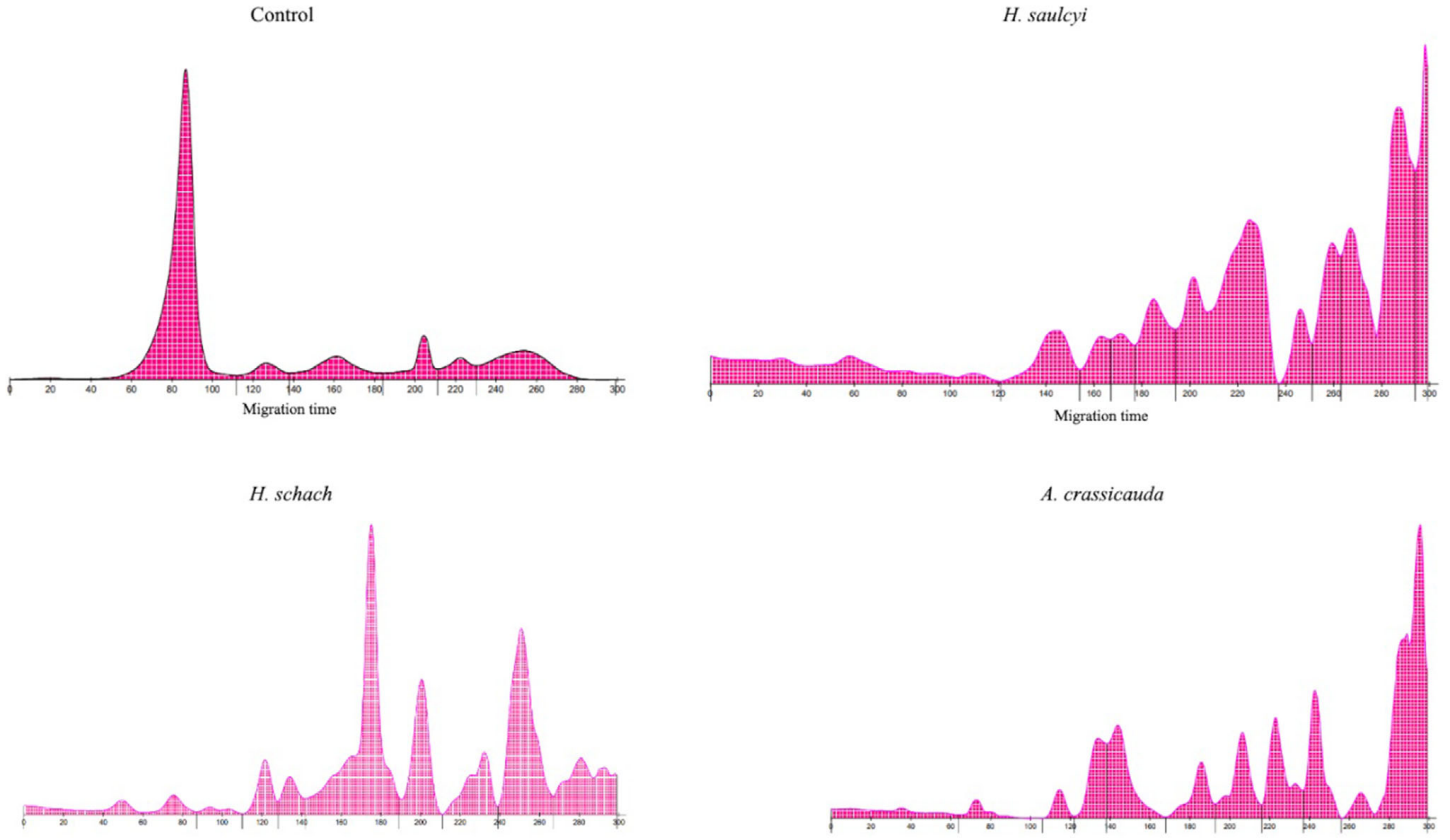
Concentration of protein in the venom of *H. schach, H. saulcyi*, and *A. crassicauda*

Protein concentrations were determined by the Biuret method. In this technique, peptide bonds in proteins produce a violet colour in the presence of alkaline copper, and the intensity of the colour is estimated with a spectrometer at 540 nm wavelength (Figure 1).

Albumin content in the venom was 11.7% (*H. saulcyi*), 5.4% (*H. schach*) and 4.4% (*A. crassicauda*). *A. crassicauda* had the lowest level of albumin. It should be noted that the albumin content in the different venoms was lower than that of the control group (61.1%) (Table 7). The protein ratio was much lower with the venom of *A. crassicauda* (0.05) followed by *H. schach* (0.06) and the highest was obtained with the venom of *H. saulcyi* (0.13). The most protein‐rich venoms were *H. saulcyi* (16.2%) and *H. schach* (16.26). The venom of *A. crassicauda* contains less proportion of proteins (14.8%) compared with those of *H. schach* and *H. saulcyi*.

**TABLE 7:** Protein fractions contained in venins of: *H. saulcyi*, *H. schach*, and *A. crassicauda*

Fractions	Control	*H. saulcyi*	*H. schach*	*A. crassicauda*
Albumin%	61.1	11.7	5.4	4.4
A/G ratio	1.57	0.13	0.06	0.05
Total protein	7.2	16.2	16.26	14.8

## 4 DISCUSSION

### 4.1 Collection of the scorpion and venom by electrical stimulation

*A. crassicauda, H. schach* and *H. saulcyi* were collected from Esfahan Farm Scorpion Company and maintained in the laboratory in containers that mimic their natural habitat. Scorpions kept in glass containers were active and healthy with a 100% survival rate. The use of a Petri dish as a water trough makes the water easily accessible to all scorpions as also reported (Nagaraj et al., 2015). According to Nagaraj et al. (2015), it is an effective and easy method to use moist cotton balls placed in a container (Candido & Lucas, 2004; Whittemore et al., 1963). Water‐efficient spraying restores soil moisture content and contributes to scorpion moulting (Rubio, 2008).

The stimulator used in this study was the one used by Nagaraj et al. (2015) for venom extraction and was easier to use than the one used by Lowe and Farrell (2011). The venom of *A. crassicauda, H. schach* and *H. saulcyi* was extracted by electrical stimulation (8 and 10 V). This voltage variability was noted by Nagaraj et al. (2015) who reported that the voltage required to stimulate scorpions varies according to species and size (Nagaraj et al., 2015). The venom of the species *Androctonus mauretanicus* and *Buthus occitanus tunetanus* was extracted by stimulation at 12 V, whereas *Parabuthus sp*., *Tityus serrulatus* and *Heterometrus gravimanus* were stimulated at 40, 12.5 and 8 V, respectively (Candido & Lucas, 2004; du Plessis, 2005; Oukkache et al., 2013; Whittemore et al., 1963). The study by Nagaraj et al. (2015) revealed that *Hottentotta rugiscutis* and *Hottentotta tamulus* secrete venom when stimulated at 8 V and that *Heterometrus swammerdami* secretes venom only at 10 or 12 V depending on the size of the species. These variations can also be related to the conditions and variability of the stimulators used. Therefore, a study conducted in Pakistan on buthids also reports that 25 V voltage is best suited for venom extraction in scorpions (Yaqoob et al., 2016). The use of an electrical signal remains an interesting technique to extract scorpion venom through the muscles of the venom gland (Lowe & Farrell, 2011).

### 4.2 Identification of scorpions

Based on the morphological characteristics such as body colour, the shape and size of pedipalps, shape of the sternum, the colour of telson and several pectine teeth, the collected scorpions were identified to the species level according to Nagaraj et al. (2015), Sureshan et al. (2007) and Veronika et al. (2013).

### 4.3 Lethality of scorpion venom

The toxicity evaluation of scorpion venom is a critical step for an efficient determination of the venom activity (Oukkache et al., 2014). The most common model for venom toxicity analyses is the LD_50_ value determination in mice (Charman et al., 2000; Charman et al., 2001; Dzikouk et al., 2002; Krifi et al., 1998). The LD_50_ calculated by probit analysis were 1.47 mg/kg for *H. saulcyi* venom, 0.85 mg/kg for *H. schach* venom and 1.70 mg/kg for *A. crassicauda* venom. Venom was obtained by electric stimulation. The venom of *A. crassicauda* thus presented the lowest toxicity and the highest toxicity was recorded for *H. saulcyi*.

Venom toxicity varies with several factors such as genus, species, members of one species, age, physiology, feeding state and region of the scorpion (Hassan, 1984; Krifi et al., 1998; Ozkan et al., 2006; Padilla et al., 2003; Theakston et al., 2003). Therefore, these parameters must be specified for any venom LD_50_ values reported.

Ismail et al. (1994a,b) found that the LD_50_ of *A. crassicauda* venom obtained by electric stimulation was 0.64 mg/kg, whereas Latoxan Laboratory reported an LD_50_ of 0.87 mg/kg for *A. crassicauda* venom obtained by the same method (Ismail et al., 1994a; Ismail et al., 1994b). However, Altinkurt and Altan (1980) reported that the LD_50_ of *A. crassicauda* venom, from the Sanliurfa region, was 11.5 mg/kg by the maceration method. The LD_50_ of scorpion venom must vary even if the venom was extracted by using a single method. Venoms from species of the *Androctonus* genus are highly toxic (Ozkan & Filazi, 2004). *A. crassicauda* venom has a 0.32 mg/kg LD_50_ when intravenously injected into mice, which makes this scorpion species one of the most toxic in the world (Bonnet, 1997; Ismail et al., 1994a; Ismail et al., 1994b). Also, Ismail (1993) reported that the LD_50_ of *Leiurus quinquestriatus* venom varies from 0.23 to 6.5 mg/kg (Ismail, 1993). Induced by the subcutaneous route, LD_50_ calculated by probit analysis was 1.1 mg/kg for venom obtained by electric stimulation (Ozkan et al., 2007).

According to Yağmur et al. (2015), the lethal assay of the *H. saulcyi* venom was 0.73 mg/kg in mice (Yağmur et al., 2015). This lethal dose 50 is lower than that obtained in the present study by the probit method.

These results confirm those of Altinkurt and Altan (1980) and Dittrich et al. (2002) which state that the same species present a range of LD_50_ values in the lethality test of venoms. The low LD_50_ obtained for *Hottentotta* species agrees with a report indicating that scorpions of the Buthidae family are of medical importance (Ozkan et al., 2011).

### 4.4 Determination of amino acids

*A. crassicauda, H. saulcyi* and *H. schach* contain 26, 30 and 31 amino acids, respectively, of which 7, 11 and 12 are in low concentrations. The different amino acids combine to form various peptides. The study conducted by Caliskan et al. (2006) on *A. crassicauda* in Turkey revealed that there are nearly 100 different amino acid residues, taking into account those present in minute quantities, and unresolved mixtures not separated by HPLC (Caliskan et al., 2006).

Scorpion venoms consist of a large library of biologically active peptides. these peptides are active against a wide range of biological targets, resulting in significative variation in their biological activity (Almaaytah & Albalas, 2014). To date, more than 40 peptides have been recognised and functionally categorised from scorpion venoms. The therapeutic and biological applications of peptides are related to their antibacterial, antifungal, antiviral, insecticidal, antimalarial, anticancer, cytolytic, anti‐inflammatory, immunomodulatory and bradykinin potentiating activities (Almaaytah & Albalas, 2014). The venom of *A. crassicauda* contains many different peptides, including toxins that are lethal to mice, confirming previous medical reports of human poisoning by bites caused by this species in Turkey (Altinkurt & Altan, 1980). The study of Caliskan et al. (2006) showed that *A. crassicauda* has more than 58 amino acids (Caliskan et al., 2006). Mohamadpour et al. (2008) also reported that *H. schach* has more than 20 chains of amino acids of different molecular weights (Mohamadpour et al., 2008).

Scorpion venom is a complex combination of proteins, peptides, amino acids and other biomolecules as well as certain minerals. This combination makes the venom a potential biological drug and therefore has a lot of therapeutic potentials to be exploited (Goudarzi & Salehi Najafabadi, 2019).

### 4.5 Concentration of proteins

Albumin content in the venom was 11.7% (*H. saulcyi*), 5.4% (*H. schach*) and 4.4% (*A. crassicauda*). *A. crassicauda* had the lowest level of albumin. The lower albumin level recorded for *A. crassicauda* indicates its higher level of purity compared with other venoms. He showed a higher level of purity followed by those of *H. schach* and *H. saulcyi*, respectively. The low presence or absence of albumin in the venom expresses its higher level of purity (Ismael et al., 2018). The protein content of venom from *H. saulcyi, H. schach* and *A. crassicauda* were 16.2%, 16.26% and 14.8%. Similar results were obtained by Ozkan et al. (2007) for *A. crassicauda* which states that protein bands of the venom sample obtained by electric stimulation were between 12 and 53 kDa (Ozkan et al., 2007).

The method adopted in this study for the maintenance of scorpions in the laboratory is efficient. The claustration device and electrical stimulation are significantly effective for the extraction of venom from scorpions of different species. The constitution of this study is important for researchers. This study could be useful for understanding the venom extraction and toxicity, the protein and amino acid diversities of three species of scorpions (*H. saulcyi, H. schach* and *A. crassicauda*). The results from this study show that *H. saulcyi* and *H. schach* contain more amino acids and protein and have higher toxicity than *A. crassicauda*. Furthermore, the venom of *A. crassicauda* has a high level of purity compared with the others. Note that toxicity, number of amino acids, protein content and degree of purity of scorpion venoms vary from one scorpion to another.

## AUTHOR CONTRIBUTION

*Formal analysis, investigation, methodology and writing‐original draft*: Ani Boghozian. *Methodology, resources, supervision, validation, visualisation, writing‐review and editing*: Habibollah Nazem. *Conceptualisation, data curation, supervision, validation, visualisation, writing‐review and editing*: Mohammad Fazilati. *Formal analysis, investigation, methodology, project administration, writing‐review and editing*: Seyed Hossein Hejazi. *Methodology, validation, visualisation, writing‐review and editing*: Mohammadreza Sheikh Sajjadieh.

## Data Availability

The data that support the findings of this study are available on request from the corresponding author.
